# Supplementary material for: Parenting and childhood obesity: Validation of a new questionnaire and evaluation of treatment effects during the preschool years
Source: PLoS One. 2021 Sep 23;16(9):e0257187. doi: 10.1371/journal.pone.0257187 (PMC8459975; doi:10.1371/journal.pone.0257187)
Supplement: S1 Table — (DOCX) [file pone.0257187.s001.docx]

**S1 Table.** Distribution of response categories of all items in the parenting questionnaire in the validation study (sub study I).

| Parenting practice | Items |  | Response options | | | | |
| --- | --- | --- | --- | --- | --- | --- | --- |
|  |  | Mean (SD) | N (%) | | | | |
|  |  |  | 1 | 2 | 3 | 4 | 5 |
| LS | My child can make me change my mind to something I first said no to (reversed). | 3.4 (0.9) | 17(3) | 68(12.2) | 202(36.2) | **234(41.9)** | **37(6.6)** |
| LS | I think it is difficult to say no to my child (reversed). | 4.1 (0.9) | 5(0.9) | 34(6.1) | 66(11.8) | **220(39.4)** | **233(41.8)** |
| ER | How I handle my child’s behavior depends on how I feel (reversed). | 3.0 (1.0) | 34(6.1) | 169(30.3) | **175(31.4)** | **137(24.6)** | 43(7.7) |
| LS | If I and my child disagree on something we end up doing what my child wants (reversed). | 4.2 (0.8) | 3(0.5) | 10(1.8) | 73(13.1) | **275(49.3)** | **197(35.3)** |
| ER | If my child doesn’t do what I say I find it hard controlling my emotions (reversed). | 4.0 (0.8) | 2(0.4) | 19(3.4) | 121(21.7) | **247(44.3)** | **169(30.3)** |
| LS | I can change my mind if my child throws a tantrum over something I have decided (reversed). | 4.2 (0.8) | 4(0.7) | 16(2.9) | 86(15.4) | **219(39.2)** | **233(41.8)** |
| ER | If I and my child want different things we end up falling out with each other (reversed). | 3.8 (0.9) | 4(0.7) | 36(6.5) | 139(24.9) | **256(45.9)** | **123(22.0)** |
| ER | If my child doesn’t listen to me I get frustrated (reversed). | 3.2 (1.0) | 26(4.7) | 119(21.3) | **172(30.8)** | **186(33.3)** | 55(9.9) |
| LS | I think it is hard to set up limits to my child (reversed). | 4.3 (0.8) | 5(0.9) | 18(3.2) | 61(10.9) | **207(37.1)** | **267(47.8)** |
| **Values in bold** indicate considerable ceiling effects (>50% of responses cluster in the higher response categories). | | | | | | | |
