# Supplementary material for: Parenting and childhood obesity: Validation of a new questionnaire and evaluation of treatment effects during the preschool years
Source: PLoS One. 2021 Sep 23;16(9):e0257187. doi: 10.1371/journal.pone.0257187 (PMC8459975; doi:10.1371/journal.pone.0257187)
Supplement: S2 Table — (DOCX) [file pone.0257187.s002.docx]

**S3 Table**. Mean differences regarding questionnaire items between children with normal weight/overweight and children with obesity (sub study I).

|  | | Normal weight /Overweight | | Obesity | |  |
| --- | --- | --- | --- | --- | --- | --- |
|  |  | N= 365 | | N= 132 | |  |
| Factor | Items | M | SD | M | SD | p-value |
| *Limit Setting* | **My child can make me change my mind to something I first said no to (reversed).** | **3.3** | **0.89** | **3.6** | **0.89** | **0.02** |
| *Limit Setting* | **I think it is difficult to say no to my child (reversed).** | **4.3** | **0.81** | **3.8** | **1.1** | **<0.001** |
| *Emotional Regulation* | **How I handle my child’s behavior depends on how I feel (reversed).** | **2.9** | **0.96** | **3.3** | **1.1** | **0.001** |
| *Limit Setting* | If I and my child disagree on something we end up doing what my child wants (reversed). | 4.2 | 0.70 | 4.1 | 0.89 | 0.07 |
| *Emotional Regulation* | If my child doesn’t do what I say I find it hard controlling my emotions (reversed). | 4.0 | 0.78 | 4.0 | 0.94 | 0.58 |
| *Limit Setting* | I can change my mind if my child throws a tantrum over something I have decided (reversed). | 4.2 | 0.82 | 4.1 | 0.95 | 0.14 |
| *Emotional Regulation* | If I and my child want different things we end up falling out with each other (reversed). | 3.8 | 0.86 | 3.8 | 0.93 | 0.83 |
| *Emotional Regulation* | If my child doesn’t listen to me I get frustrated (reversed). | 3.2 | 1.00 | 3.3 | 1.2 | 0.72 |
| *Limit Setting* | **I think it is hard to set up limits to my child (reversed).** | **4.4** | **0.76** | **3.9** | **0.98** | **<0.001** |
| *Mean*  *Scores* | **LIMIT SETTING** | **4.1** | **0.58** | **3.9** | **0.76** | **0.005** |
|  | EMOTIONAL REGULATION | 3.5 | 0.64 | 3.6 | 0.78 | 0.25 |
| Items **in bold** presented significant differences in mean scores between children with normal weight/overweight & children with obesity | | | | | | |
