# Supplementary material for: Parenting and childhood obesity: Validation of a new questionnaire and evaluation of treatment effects during the preschool years
Source: PLoS One. 2021 Sep 23;16(9):e0257187. doi: 10.1371/journal.pone.0257187 (PMC8459975; doi:10.1371/journal.pone.0257187)
Supplement: S3 Table — (DOCX) [file pone.0257187.s003.docx]

**S4 Table.** Effects of treatment group, time and group-by-time interaction on the four parenting practices outcomes-maternal Limit Setting and Emotional Regulation, and paternal Limit Setting and Emotional Regulation.

|  | Intercept ^a^ (SE) | Booster by time ^b^ | p-value* | No-Booster by time ^c^ | p-value* | Time ^d^ | p-value** |
| --- | --- | --- | --- | --- | --- | --- | --- |
|  |  | *(95% CI)* |  | *(95% CI)* |  |  |  |
| Mother LS | 3.95 | -0.001 *(-0.026 to 0.024)* | 0.917 | -0.001 *(-0.024 to 0.022)* | 0.934 | **0.018** | **0.011** |
| Mother ER | 3.70 | 0.025 *(-0.002 to 0.051)* | 0.07 | -0.008 *(-0.033 to 0.016)* | 0.52 | 0.009 | 0.25 |
| Father LS | 4.09 | -0.018 *(-0.041 to 0.005)* | 0.11 | 0.000 *(-0.022 to 0.022)* | 0.98 | 0.014 | 0.04 |
| Father ER | 3.80 | **-0.025 *(-0.050 to 0.000)*** | **0.054** | **-0.031 *(-0.055 to -0.006)*** | **0.01** | **0.016** | **0.03** |
| ^a^ Intercept: predicted value at baseline for ST  ^b^ Interaction between PGB and time (months). ST as reference  ^c^ Interaction between PGNB and time (months). ST as reference  ^d^ Coefficient for time (months) for ST  *p-value for difference between groups (group by time interaction) with ST as reference  **p-value for change from baseline for ST | | | | | | | |
