# Supplementary material for: Parenting and childhood obesity: Validation of a new questionnaire and evaluation of treatment effects during the preschool years
Source: PLoS One. 2021 Sep 23;16(9):e0257187. doi: 10.1371/journal.pone.0257187 (PMC8459975; doi:10.1371/journal.pone.0257187)
Supplement: S4 Table — Path coefficients to examine the mediating effect of parenting practices (mothers’ and fathers’) on treatment effects on the primary outcome (changes in child weight status at 12 months post-baseline). (DOCX) [file pone.0257187.s004.docx]

**S5 Table.** **Mediation model.** Path coefficients to examine the mediating effect of parenting practices (mothers’ and fathers’) on treatment effects on the primary outcome (changes in child weight status at 12 months post-baseline).

|  |  | **Changes in child weight status (12 months post-baseline)** | |
| --- | --- | --- | --- |
|  |  | **Indirect effect**  **a*b** | **95% CI *** |
| Maternal Limit Setting ^1^  (n=110) | Treatment group/PGNB ^a^ | 0.0003 | -0.001 to 0.002 |
|  | Treatment group/PGB ^a^ | 0.0005 | -0.001 to 0.003 |
| Maternal Emotional Regulation ^2^  (n=110) | Treatment group/PGNB ^a^ | 0.0005 | -0.001 to 0.003 |
|  | Treatment group/PGB ^a^ | -0.0002 | -0.003 to 0.002 |
| Paternal Limit Setting ^3^  (n=101) | Treatment group/PGNB ^a^ | 0.0002 | -0.001 to 0.002 |
|  | Treatment group/PGB ^a^ | -0.0001 | -0.001 to 0.002 |
| Paternal Emotional Regulation ^4^  (n=100) | Treatment group/PGNB ^a^ | -0.0002 | -0.003 to 0.002 |
|  | Treatment group/PGB ^a^ | -0.0006 | -0.003 to 0.002 |
| ^a^ ST as reference group  ^1^ Mediation model 1: Changes in maternal Limit Setting is the proposed mediator  ^2^ Mediation model 2: Changes in maternal Emotional Regulation is the proposed mediator  ^3^ Mediation model 3: Changes in paternal Limit Setting is the proposed mediator  ^4^ Mediation model 4: Changes in paternal Emotional Regulation is the proposed mediator  *Mediation established if 0 is NOT included in the CIs at the 95% level. | | | |
