# Supplementary material for: Parenting and childhood obesity: Validation of a new questionnaire and evaluation of treatment effects during the preschool years
Source: PLoS One. 2021 Sep 23;16(9):e0257187. doi: 10.1371/journal.pone.0257187 (PMC8459975; doi:10.1371/journal.pone.0257187)
Supplement: S1 Fig — Mean scores in both parenting practices were higher than average. (DOCX) [file pone.0257187.s005.docx]

**S1 Fig.** Distribution of mean scores in Limit Setting and Emotional Regulation in the validation study (sub study I). Mean scores in both parenting practices were higher than average.
