# Supplementary material for: Parenting and childhood obesity: Validation of a new questionnaire and evaluation of treatment effects during the preschool years
Source: PLoS One. 2021 Sep 23;16(9):e0257187. doi: 10.1371/journal.pone.0257187 (PMC8459975; doi:10.1371/journal.pone.0257187)
Supplement: Attachment — Submitted filename: PONE Journal Review.pdf [file pone.0257187.s007.pdf]

PLOS ONE Journal Review

Ms. Title: "Parenting and childhood obesity: Validation of a new questionnaire and evaluation of treatment effects during the preschool years"

Reviewer Comments for Authors

In this study, the authors examined the effects of an RCT that targets obesity in preschoolers through enhancing parenting skills in mothers and fathers. They also developed and validated a novel parenting questionnaire to examine the efficacy of the RCT on targeted parenting skills. This is an important topic, particularly in the context of preschool-aged children. The overall paper is well-written. However, there are major concerns in how models were tested that must be addressed.

Abstract

1. BMI-Z should be changed to BMI SDS, as was used throughout the study.

Introduction

2. P. 4, ln. 45: Five constructs of parenting practices were hypothesized to be aligned with the treatment targets. While there is evidence to support the importance of these parenting constructs on child outcomes, the authors do not provide evidence of how they are relevant to preschoolers' weight status. Other than limit-setting, a known predictor of child BMI, the other constructs are too general to imply how they relate. For instance, were these measured explicitly in the feeding/mealtime context?

3. To that same end, the reason for including emotion regulation is also unclear. Studies have suggested the association between ER and obesity. However, the introduction does not explain how this was relevant to parenting. Specifically, how does this operate in the context of parenting that impacts childhood obesity? It would be helpful to include 'parent' when referring to the emotion regulation construct, given the strong and more commonly assessed relation between child ER and obesity.

4. It would be helpful for a brief description of the treatment 'booster sessions' in the introduction.

5. P. 5: Aim 2 in study II focuses on changes in parenting practices due to the intervention. What about a direct relation between the RCT and changes in weight status since the intervention was intended to target obesity? Aim 3 does partially examine this link, but not much detail is provided.

Methods

Study I

6. P. 9 1. What is the difference between schools and preschools? Do you mean schools and classrooms within each school, or were these different grade-levels? Please clarify.

7. Also, given that participants were recruited in schools were there differences in the factor analyses by school? If so, examining these analyses in a two-level framework may be warranted.

8. P. 9 How does the clinical sample differ from the school sample? Were they recruited differently, or were those categorized with obesity status assigned to the clinical group and the remaining school sample? Was the 177 clinical sample in Study I the same sample randomized in Study II? This was not clear in the text.

Results

9. What was the rationale for combining the normal weight and overweight categories, especially with evidence suggesting that preschoolers with overweight status are at higher risk? Models should be stratified by the three groups.

10. Table 1 the mean BMI SDS was -0.3 (SD=1.2) for the school sample, suggesting that some of the children's weight may fall below the normal weight status. Were there no children with underweight status? If not, what was there a process for dropping those children during the screening phase?

Study I
11. P. 16 The authors noted that 'mothers represented a majority of the sample (82%)', suggesting that parenting scales were valid for mothers, but not definitively for fathers given the limited sample. Why were the analyses in Study I not conducted between mothers and fathers?

12. P. 17 Two parenting scales, limit setting and emotion regulation, confirmed factor loadings >0.4. However, these findings differ from the previously stated findings on P. 8, indicating that only monitoring and limit setting scales met the recommended levels. Please clarify the difference.

13. P. 19 Were relevant covariate controlled for in the correlation analyses with parenting scales? This is important given the evidence at both the parent and child levels.

Study II
14. P. 19 Please define how 'foreign background' was assessed? Were they expected to differ in parenting or child outcomes? This should be introduced earlier with a rationale.

15. P. 20 Were the linear mixed models adjusted for relevant covariates?

16. P. 21 What were the estimates for the direct effects of parenting behaviors or treatment effects on BMI SDS in the mediation models? Did mediation models adjust for relevant covariates?

Discussion

17. Overall, the discussion taps into several different areas. However, the explanations about the RCT effects should be modified. For example, this study found that mothers increased limit-setting overall, but not as a result of the intervention. This suggests that there was either a naturally-occurring increase in behaviors overtime or a third modifying variable. As for fathers, emotion regulation significantly changed for fathers in the two parenting intervention groups, assuming that the standard treatment was used as the comparison group (not a parenting intervention aimed at similar components of parenting as the other groups). However, this change indicated that fathers in the treatment group had a decline in emotion regulation (parenting tx = poorer emotion regulation). That said, no causal pathways were supported.

18. P. 27, ln. 539 The authors suggest that this study addresses limitations in obesity research by using a more diverse SES sample. This is an overly ambitious statement given the limited details about the socio-demographic characteristics of the sample provided in the introduction and results. Additionally, no statistical testing (e.g., moderation) was performed for any conclusions about the differences of these various groups.

19. P. 27 Why was the power analysis only provided for treatment effects on child BMI SDS? Particularly since none of this study's aims/hypotheses examined the direct effect of treatment on child weight status.

Minor Points

20. In the methods and results, how the content of study 1 and 2 are presented is somewhat confusing to follow. Instead of switching back and forth between studies within each section, it may be easier to first present study 1 (methods, data analyses, and results) and then study 2 (methods, data analyses, and results).

21. In figure 3, the PGB and ST lines should reflect more distinguished differences (e.g., change one to a shade of gray or thicker lines).
