# Supplementary material for: Parenting and childhood obesity: Validation of a new questionnaire and evaluation of treatment effects during the preschool years
Source: PLoS One. 2021 Sep 23;16(9):e0257187. doi: 10.1371/journal.pone.0257187 (PMC8459975; doi:10.1371/journal.pone.0257187)
Supplement: Attachment — Submitted filename: R2R PLOS Revision.pdf [file pone.0257187.s008.pdf]

Cover and response letter to comments from the reviewers. PONE-D-20-28940
Title: Parenting and childhood obesity: Validation of a new questionnaire and evaluation of treatment effects during the preschool years

Dear Editor-in-Chief, Dr. Heber,

We thank the editor for giving us the opportunity to improve our manuscript. We have responded to all the reviewers' comments, which have been constructive and very helpful. We believe the manuscript is much stronger as a result of this revision. Thank you, in advance, for your review.

Sincerely,
Paulina Nowicka and co-authors

<u>Response letter</u>

Reviewer 1 (R1)

R1	Reviewer #1: Important note: This review pertains only to 'statistical aspects' of the study and so 'clinical aspects' [like medical importance, relevance of the study, 'clinical significance and implication(s)' of the whole study, etc.] are to be evaluated [should be assessed] separately/independently. Further please note that any 'statistical review' is generally done under the assumption that (such) study specific methodological [as well as execution] issues are perfectly taken care of by the investigator(s). This review is not an exception to that and so does not cover clinical aspects {however, seldom comments are made only if those issues are intimately / scientifically related & intermingle with 'statistical aspects' of the study}. Agreed that 'statistical methods' are used as just tools here, however, they are vital part of methodology [and so should be given due importance]
Author response	We thank the reviewer for their comments and suggestions. The points have now been addressed.
R1	COMMENTS: Your ABSTRACT is well drafted but assay type. Please note that it is preferable [refer to item 1b of CONSORT checklist 2010: Structured summary of trial design, methods, results, and conclusions] to divide the ABSTRACT with small sections like 'Objective(s)', 'Methods', 'Results', 'Conclusions', etc. which is an accepted practice of most of the good/standard journals [including PLOS-ONE]. It will definitely be more informative then, I guess, whatever the article type may be.
Author response	We thank the reviewer for the suggestion. We revised the Abstract accordingly (p.2).
R1	Unfortunately, there are several questions [naturally arising in mind] about this article. Few important/vital ones [not possible to cover all] are described/given below. First let us refer to lines 198-202, section on 'Sample size' [as vital for present statistical review] which quotes reference by Kleber et al., 2009 {'Power calculations were based on the primary outcome, child BMI SDS (Body Mass Index Standard Deviation Score) (Kleber et al., 2009)}. Are you referring to reference number 19? But it is said in line 30 that their study (reference 19) is

	'without a control condition' then how can 'power calculations are based' on this study or do you want to say that 'the primary outcome, child BMI SDS (Body Mass Index Standard Deviation Score)' this term is used for first time by them or the term is taken from this reference? Clarify the purpose of quoting the article. Note that "Kleber" is second author of paper quoted as 19 and instead of [19], quoting (Kleber et al., 2009) is not correct at all. Please follow the standard practice of quoting reference(s)
Author response	Thank you for this comment. We have now corrected the reference using standard practices of quoting references, also according to the journal standards (pp. 13-14, lines 240-246). Moreover, our response to the next comment clarifies the power calculations in the RCT.
R1	Further, it is said that '…. adjusting for a dropout rate of 21%'. Are following references (namely Ek, Chamberlain, et al., 2015; West et al., 2010) are for the (odd) figure of 21%? Account given in section on 'Sample size' (lines 198-202) 'Seventy-five children needed to be included in each of the treatment approaches (ML program and standard treatment) in order to identify a difference in BMI SDS between the groups at 12-months post-baseline' is/are inadequate [not sufficient at all] and therefore, of no use { to identify what amount of difference in BMI SDS?}.
Author response	This point has now been clarified in the manuscript in pp. 13-14, lines 240-246.<br><br>The text now reads:<br>*Sample size: The sample size calculation for the ML study was based on data from a treatment study in Germany [51]. On the basis of power calculations, 75 children were needed in each treatment (parent-only and ST adjusted for dropout) to detect a difference of 0.3 BMI z score (0.5 SD) with 85% power at 12 months' follow-up. The calculations included an adjustment for a dropout rate of 21%, based on data from a similar study of obesity treatment focusing on parenting [52]. The sample size calculation has been described previously [24]. (*pp. 13-14, lines 240-246)
R1	Though measures/tools used are appropriate, most of them [example: Child Feeding Questionnaire (CFQ), the Lifestyle Behaviour Checklist (LBC), etc.] yield data that are in [at the most] 'ordinal' level of measurement [and not in ratio level of measurement for sure {as the score two times higher does not indicate presence of that parameter/phenomenon as double (for example, a Visual Analogue Scales VAS score or say 'depression' score)}]. Then application of suitable non-parametric test(s) is/are indicated/advisable [even if distribution may be 'Gaussian' (i.e. normal)].
Author response	We thank the reviewer for this suggestion. We considered the ordinal nature of our data collected through valid obesity-related instruments, and we applied nonparametric tests, i.e., nonparametric correlations through Spearman's rho. This point has been clarified in the Methods section:<br><br>*Nonparametric correlation coefficients (Spearman's rank correlation coefficient) were computed to quantify the strength and direction of associations between the parenting questionnaire and the CFQ and LBC. (*p. 16, lines 300-302)<br><br>Table 3 in the manuscript has been modified accordingly (p. 22, lines 407-409).

	The findings and conclusions drawn based on the nonparametric tests are largely the same as before (limit setting was additionally found to correlate with monitoring in the expected positive direction, which is consistent with the conclusion of the analysis), and the text in results has been modified accordingly:<br><br>*Both LS and ER correlated weakly with CFQ restriction, pressure to eat, and monitoring (no coefficient exceeded 0.15, absolute number). In particular, parents who reported higher scores on LS also reported higher scores in monitoring and lower scores in restriction. Parents reporting higher scores in ER reported lower scores in pressure to eat and higher scores in monitoring.*<br>*Regarding correlations with LBC factors, parents who reported high levels of LS and ER also reported lower scores in child problematic behaviors related to obesity (no coefficient exceeded 0.25, absolute number), and higher scores in confidence in tackling those problems (no coefficient exceeded 0.33, absolute number). (*p. 21, lines 396-404)
R1	Now refer to lines 228-234<br>{Statistical Analyses - Sub study I: Differences between subsamples were examined using independent samples t-test for continuous variables and chi-squared test for categorical variables. Background characteristics were compared across the school and clinical samples using one-way Analysis of Variance (ANOVA) (for continuous variables) and chi-square test (for categorical variables).}<br>Use Mann-Whitney U test in place of independent samples t-test. Note that we use Analysis of Variance (ANOVA) [for continuous variables] to compare three or more groups {not to compare two groups]. You seem to compare two groups [the school and clinical samples] only.<br>Following note is from one standard text-book on Biostatistics:<br>When only two groups are to be compared {ex. Women and Men}, we use 't' test for two independent groups [non-parametric equivalent to unpaired 't' test is Mann-Whitney 'U' test] and not ANOVA 'F' Although 'F' and 't' are mathematically related/equivalent [square of 't' is exactly equal to 'F' if (mistakenly) calculated for two groups], logic/philosophy (and so underlying assumptions) behind their development and algorithms used for estimation of test statistic are different. Mind you that they are applicable in different situations.<br>Choice of test will not depend on 'Type of variable' or 'Level of measurement' of parameter/background characteristic [line 231 or 256] under consideration. {Non-parametric equivalent of one-way ANOVA 'F' is 'Friedman's test'}. If treatment group are now [i.e. your are considering] three conditions [as PGB: Parent group with booster sessions; PGNB: Parent group without booster sessions; ST: Standard Treatment], clarify. Kindly remember that this is a scientific/academic document and so all details should be clearly communicated.
Author response	We thank the reviewer for giving us the opportunity to clarify the descriptive analyses in study I. We indeed performed independent samples t-tests to compare the two independent groups in study I, namely the (pre)school and clinical samples. Thus, we removed the additional sentence referring to ANOVA in this section. We agree with the reviewer that the choice of test will not depend on the type of variable or level of measurement of background characteristics. However, we performed descriptive analyses in order to describe the sample, since it is composed by two different sub samples. Therefore, we provide the mean (SD) and n (%) –for continuous and categorical variables, respectively– to enhance understanding of the sample characteristics. These points have now been clarified in the text and the changes can be tracked in p. 15 lines 281-284.<br>The text now reads:

	*Differences between subsamples were examined using independent samples t-test for continuous variables and chi-squared test for categorical variables. Descriptive characteristics are presented using means (standard deviations) and n (%), for continuous and categorical variables, respectively. (*p. 15 lines 281-284)<br><br>In addition, we used non-parametric Mann-Whitney U test in order to confirm the differences in parenting practices between children with obesity and without obesity, which are presented using mean (SD) and have been tested through parametric t-test.<br><br>The text reads:<br>*In addition, mean differences in parenting items between parents of children with obesity and parents of children without obesity were investigated using parametric t-tests (due to the ordinal nature of the parenting questions, the findings were confirmed using non-parametric Mann-Whitney U test and Wilcoxon signed rank sum test)*. (p. 16, lines 302-306)<br><br>As for study II, we used one-way ANOVA to compare the three treatment groups, namely ST, PGB and PGNB. (p. 16, lines 308-309)<br><br>The text that was removed is:<br>*Background characteristics were compared across the school and clinical samples using one-way Analysis of Variance (ANOVA) (for continuous variables) and chi-square test (for categorical variables).*
R1	In 'Abstract' you say "A merged school/clinical sample (n=558, 82% mothers) was used for the factorial and construct validation of a new parenting questionnaire. Changes in parenting were evaluated using data from the More and Less Study, a randomized controlled trial (RCT) with 174 children (mean age=5 years, mean BMI-Z =3.0) comparing a parent support program (with and without booster sessions) and standard treatment.". Further, you say "We administered the new questionnaire to the RCT participants.". Is not that confusing? Where and when this RTC (line 39-40: an RCT for obesity treatment among preschoolers, the More and Less study (ML study) [23].) was conducted? As said in lines 54-6 [The evaluation includes two stages: performing a validation study on a new questionnaire on parenting practices (Sub study I) and assessing parenting practices using data from the ML study RCT (Sub study II).] it seems that you have used entirely different samples for these two stages {Sub study I & Sub study II}. Is that true? It is not clear 'whether n=558 or n=174' for this study [how can different stages of the same can use different samples?]. Again, remember that this is a scientific/academic document and so all details should be clearly communicated. Do not confuse readers. I found many things confusing in this article. Please do not take readers for granted {that they will understand what is there in your mind}. It may be 100% correct, but need to (should) be communicated clearly.
Author response	We have clarified the two different objectives of the paper (mirroring study I and study II, and their respective samples) in the Abstract. In addition, we have clarified in the manuscript (under the Methods section) that the clinical sample, which was used to enrich the school

	sample in the validation was drawn from baseline data of the More and Less study (p. 11, lines 184-187). The longitudinal and randomized design facilitated the evaluation of changes in parenting practices as described in study II.<br><br>The text reads:<br><br>*Clinical sample (ML study): Baseline data from the ML study were used, i.e. the questionnaire on parenting, the CFQ, the LBC and information about sociodemographic background. Since the ML study is the focus of study II, its experimental and longitudinal design is described in the next section. (*p. 11, lines 184-187)<br><br>Moreover, we agree with the reviewer that the wording of the text, i.e. “sub study I” and “sub study II” may be misleading and confusing. Therefore, we now refer to the manuscript as “paper” (p. 6, line 69), and throughout the text, “sub study I” and “sub study II” have been replaced by “study I” and “study II”, respectively.
R1	Refer to Table 3 [Correlations between Limit Setting/Emotional Regulation and the CFQ and LBC. Mean group differences in Limit Setting/Emotional Regulation between children with obesity and children without obesity (normal weight/overweight)]. In this context note that [mainly because you have used ‘n.s.’ in this table],<br>Statistical test usually used to assess significance of Pearson’s ‘Correlation coefficient (r)’ is ‘t’ [where t = { r √ [(n-2) / (1-r2)] }for df=n-2, n is sample size] and here Ho is that the population/standard value of ‘r’ is zero. You need r=0.878 to be significant at 5% when n=5 but you need r=0.273 if n=50 & you need only r=0.088 if n=500. ‘P-value’ heavily depends on sample size. Therefore, it is customary to use the (available in most text books on ‘Biostatistics’ or on ‘www/net’) following guidelines for interpreting positive or negative correlations (and do not rely only on corresponding ‘P’-value but also consider an absolute value of ‘Correlation coefficient’). [This argument is equally applicable to non-parametric Spearman’s ‘Correlation coefficient (ρ)’ as well.]
Author response	We agree with the reviewer that the absolute coefficient is most relevant in interpreting the aforementioned correlations. Therefore, we have now provided all relevant values in Table 3 (p. 22, lines 407-409), regardless of their assigned level of statistical significance. We would like to point out that when describing these findings, we have not added any p-value, rather we have focused on describing the direction of correlations while providing the highest absolute number for the coefficients. Based on the reviewer’s suggestion we modified the results text to highlight the direction of correlations and the relatively weak nature of those by providing the highest absolute number for the coefficients (p. 21, lines 396-404).<br><br>The text reads:<br>*Both LS and ER correlated weakly with CFQ restriction, pressure to eat, and monitoring (no coefficient exceeded 0.15, absolute number). In particular, parents who reported higher scores on LS also reported higher scores in monitoring and lower scores in restriction. Parents reporting higher scores in ER reported lower scores in pressure to eat and higher scores in monitoring.*

	Regarding correlations with LBC factors, parents who reported high levels of LS and ER also reported lower scores in child problematic behaviors related to obesity (no coefficient exceeded 0.25, absolute number), and higher scores in confidence in tackling those problems (no coefficient exceeded 0.33, absolute number). (p. 21, lines 396-404)
R1	There are two more questions regarding Table three.<br>1. You have tested mean group differences in 'Limit Setting & Emotional Regulation' between children with obesity and children without obesity separately by 'Individual samples t-test' which is perfectly alright but why 'Paired samples t-test' is applied for TOTAL? Is that correct (are you dealing with pairs now)? How? Please explain {this question is due to footnote}.<br>2. Why references are quoted in footnote [a Validation study of the CFQ in Sweden [35], b Validation study of the LBC in Sweden [33]]. This implies that 'r' values are reported from these references. Is that so? Please explain
Author response	1. We used individual samples t-test to examine the mean group difference in limit setting and emotional regulation between children with obesity and without obesity. However, we used paired samples t-test since this analysis refers to the same (total) sample and it aims to check for differences between the overall limit setting and the overall emotional regulation levels among the same sample of children. This point has now been clarified in the second footnote of Table 3 (p. 22, lines 407-409).<br>2. The references in the footnote of Table 3 refer to the validation studies in Sweden, which determined the items to be considered in the calculation of the mean score for the CFQ and LBC subscales. This has now been clarified in the last two footnotes of Table 3 (p. 22, lines 407-409).
R1	I refrain from giving adverse comments on many other points in manuscript however, I definitely feel [am almost sure] that this study has potential. Presentation of (hard achieved) material is poorly done. Re-drafting avoiding confusion is recommended.
Author response	We thank the reviewer for their valuable comments which have helped us improve the manuscript.

Reviewer 2 (R2)

R2	In this study, the authors examined the effects of an RCT that targets obesity in preschoolers through enhancing parenting skills in mothers and fathers. They also developed and validated a novel parenting questionnaire to examine the efficacy of the RCT on targeted parenting skills. This is an important topic, particularly in the context of preschool-aged children. The overall paper is well-written. However, there are major concerns in how models were tested that must be addressed.
Author response	We thank the reviewer for their comments and suggestions. In the following responses we have addressed and clarified several points concerning the submitted manuscript.
R2 Abstract	BMI-Z should be changed to BMI SDS, as was used throughout the study.
Author response	We have now applied the term BMI SDS consistently throughout the text including the abstract.

R2 Introduction	P. 4, ln. 45: Five constructs of parenting practices were hypothesized to be aligned with the treatment targets. While there is evidence to support the importance of these parenting constructs on child outcomes, the authors do not provide evidence of how they are relevant to preschoolers' weight status. Other than limit-setting, a known predictor of child BMI, the other constructs are too general to imply how they relate. For instance, were these measured explicitly in the feeding/mealtime context?
Author response	Thank you for giving us the opportunity to elaborate on the evidence-based parenting skills composing the More and Less parenting program. We have now clarified in the Introduction (p. 6, lines 52-67) that the program and the parenting skills it addresses refer to existing and integrative parenting programs, which have been successful in the field of developmental psychology regarding child behavioral problems. Therefore, the five constructs of parenting practices are not only aligned with the treatment targets, but are also explicit targets of the parenting program.<br><br>The revised text reads:<br>[…] *The parenting program draws on the Oregon Model of Behavior Family Therapy, which emphasizes a core set of evidence-based parenting skills, i.e. encouragement, monitoring, limit-setting strategies, positive involvement, problem solving, and emotional regulation [26-28]. Thus, the parenting program focuses on important aspects of demandingness (e.g. limit setting) and responsiveness (e.g. emotional regulation), which compose the favorable authoritative parenting style with regard to childhood obesity [9, 29]. While parenting programs unrelated to obesity have addressed child behaviors in randomized studies successfully [30, 31], they have rarely been applied in the field of early childhood obesity [21]. The relevance of parenting skills in early childhood obesity, however, has been highlighted in observational studies [32, 33]. In addition to the parenting practices, the ML program offers developmentally appropriate information around healthy lifestyles (nutrition and physical activity) [23]. To evaluate the central parenting components of the ML program (encouragement, monitoring, limit-setting strategies, positive involvement, problem solving, and emotional regulation) a valid user-friendly tool was required. Given the lack of appropriate evaluation instruments, we developed a questionnaire to assess changes in parental behaviors after participating in the ML program.* (p. 6, lines 52-67)
R2 Introduction	To that same end, the reason for including emotion regulation is also unclear. Studies have suggested the association between ER and obesity. However, the introduction does not explain how this was relevant to parenting. Specifically, how does this operate in the context of parenting that impacts childhood obesity? It would be helpful to include 'parent' when referring to the emotion regulation construct, given the strong and more commonly assessed relation between child ER and obesity.
Author response	Parental emotional regulation is an integral component of the Parent Management Training–Oregon model (abbrev. PMTO), which underlies the Oregon Models of Behavior Family Therapy, as cited in the manuscript (Dishion, Forgatch, Chamberlain, & Pelham, 2016). These models provide the conceptual basis of the pure parenting component of the More and Less study, which also incorporated a lifestyle component in order to be applied in the field of early obesity treatment. In other words, the lifestyle component offers the relevant and developmentally appropriate information regarding changes in nutrition and physical activity, while the parenting component offers actionable advice and training in parenting, which can facilitate lifestyle changes at the family level. Please see the response and tracked manuscript changes in the previous response (p. 6, lines 52-67).

	Thank you for the suggestion to include "parent" when referring to ER, as this is the focus of the paper. We have clarified this point in the Results (p. 20, lines 380-384).<br><br>The text in Results reads:<br>*Based on the content of their respective items (factor loadings >0.4), these factors were labelled 1) Limit Setting (LS), and 2) Emotional Regulation (ER). Therefore, the new questionnaire was titled "Emotions and Communication in Parenting (ECoP)". From now on, the abbreviations LS and ER will be used to describe the results, referring to parent limit setting and parent emotional regulation respectively.* (p. 20, lines 380-384)<br><br>As for the discussion, we have clarified this in the first paragraph (p. 25, lines 455-456). Otherwise, we believe that the use of active voice in the discussion makes it clear that emotional regulation refers to parents' – and not children's – capacity to regulate their own emotions.<br><br>The text in Discussion reads:<br>*The first dimension assesses parents' limit setting and the second dimension assesses parents' ability to regulate their own emotions.* (p. 25, lines 455-456)
R2 Introduction	It would be helpful for a brief description of the treatment 'booster sessions' in the introduction.
Author response	We have clarified this point in the Introduction (p. 5, lines 41-44).<br><br>The text reads:<br>*The ML study compares a parenting program with and without booster sessions (follow-up phone calls provided after the program, averaging 4 phone calls per family) and standard treatment; the primary study outcome is change in child weight status (body mass index standard deviation score, BMI SDS).* (p. 5, lines 41-44)
R2 Introduction	P. 5: Aim 2 in study II focuses on changes in parenting practices due to the intervention. What about a direct relation between the RCT and changes in weight status since the intervention was intended to target obesity? Aim 3 does partially examine this link, but not much detail is provided.
Author response	The direct link between the parenting program and changes in weight status (the primary outcome) has been examined through an RCT and reported by Ek et al. (2019), which is extensively cited throughout the text. We have now clarified the findings from this study in relation to changes in child weight status in the Introduction (p. 5, lines 44-47) and in the Hypotheses for study II, to provide a reference for the respective findings on changes in parenting practices (p. 7, lines 90-94).<br><br>The text in Introduction reads:

	The primary findings at 1 year post-baseline showed a greater decrease in child weight status among families randomized to the parenting program with booster sessions, compared to standard care and the parenting program without booster sessions [24]. (p. 5, lines 44-47)<br><br>The text in Introduction reads:<br>*Our hypotheses were informed by the primary findings of the ML study, which showed that children of families who were randomized to the parenting program –in particular, those who received the additional booster sessions– decreased their weight status more compared to children of families in standard treatment [23, 24].* (p. 7, lines 90-94)
R2 Methods study I	P. 9 1. What is the difference between schools and preschools? Do you mean schools and classrooms within each school, or were these different grade-levels? Please clarify.
Author response	Thank you for letting us clarify this point. For simplicity, we used 'school sample' to refer to a sample composed of both schools and preschools. In Sweden children go to preschool until they are five years old; at age six they go to school. The first year in the school setting is a preparation year, so children do not start first grade until they are seven years old. Preschools and schools are separate organizations. Thus, to be able to include parents of six-year-old children in our study, we needed to reach out to both preschools and schools to distribute the questionnaires. We now clarify this in the manuscript:<br><br>*School sample: To reach the parents of 4-5 year olds, thirty preschools were selected; to reach the parents of 6 year olds, fifteen schools were selected.* (p. 11, 176-177)
R2 Methods study I	Also, given that participants were recruited in schools were there differences in the factor analyses by school? If so, examining these analyses in a two-level framework may be warranted.
Author response	Thank you for this comment. Preschools/schools were used as effective channels for reaching out to parents of children and, thus, identifying differences between those was outside the scope of study I. In addition, preschools/schools were selected from areas with low, middle, and high levels of obesity, as explained in Methods (p. 11, lines 176-179), which was relevant to the aims of study I and the paper overall.<br><br>The text in Methods reads:<br>*To reach the parents of 4-5 year olds, thirty preschools were selected; to reach the parents of 6 year olds, fifteen schools were selected. The preschools and schools were selected from different areas across Stockholm County with low, medium, and high prevalence of obesity [38].* (p. 11, lines 176-179)
R2 Methods study I	P. 9 How does the clinical sample differ from the school sample? Were they recruited differently, or were those categorized with obesity status assigned to the clinical group and the remaining school sample? Was the 177 clinical sample in Study I the same sample randomized in Study II? This was not clear in the text.

Author response	We have now clarified this point in p. 11, lines 184-187. In addition, Table 1 (p. 19, lines 368-369) shows the added value of the clinical sample, since the school samples included only one child with obesity.<br><br>The text reads:<br>*Clinical sample (ML study): Baseline data from the ML study were used, i.e. the questionnaire on parenting, the CFQ, the LBC and information about sociodemographic background. Since the ML study is the focus of study II, its experimental and longitudinal design is described in the next section.* (p. 11, lines 184-187)
R2 Results study I	What was the rationale for combining the normal weight and overweight categories, especially with evidence suggesting that preschoolers with overweight status are at higher risk? Models should be stratified by the three groups.
Author response	We agree with the reviewer that preschoolers with overweight are at higher risk for developing obesity. However, we decided to combine the normal weight and overweight categories, and retain all children classified with obesity in a separate group, since early childhood obesity has been the focus of the studies presented in the paper. In addition, the obesity status was based on measurements performed in the context of the ML study, since almost the entire sub sample of children with obesity came from this clinical sample at baseline (see Table 1). Thus, our aim was to differentiate between children with extreme weight status, present early in life –which predisposes to increasing weight gain trajectories over the lifetime (Buscot et al., 2018)– and not necessarily define a trend across groups of increasing weight status. In addition, research on obesity-related child eating behaviours, which may influence food parenting (Webber, Cooke, Hill, & Wardle, 2010), has identified differences between clinical samples who are treatment-seeking and clinical samples in the community (Croker, Cooke, & Wardle, 2011). This point has been clarified in the manuscript (p. 11, lines 190-192).<br><br>The text reads:<br>*Calculations of the BMI SDS and the classification of children according to their obesity status (children with obesity and without obesity) –in line with the focus of this paper on childhood obesity per se– were based on the criteria by Cole and Lobstein (41).* (p. 11, lines 190-192)
R2 Results study I	Table 1 the mean BMI SDS was -0.3 (SD=1.2) for the school sample, suggesting that some of the children's weight may fall below the normal weight status. Were there no children with underweight status? If not, what was there a process for dropping those children during the screening phase?
Author response	We have now clarified this point in pp. 11-12, lines 192-195. Eighteen (18) children in the school sample were classified in the underweight category and, thus, they were included in the non-obesity category.<br><br>The text reads:<br>*Moreover, 18 children were classified in the underweight category, according to age- and gender-specific criteria for thinness among children (child weight status equivalent to BMI<17) [42], and they were included in the non-obesity category.* (pp. 11-12, lines 192-195)

R2 Results study I	P. 16 The authors noted that 'mothers represented a majority of the sample (82%)', suggesting that parenting scales were valid for mothers, but not definitively for fathers given the limited sample. Why were the analyses in Study I not conducted between mothers and fathers?
Author response	We agree with the reviewer that this is a relevant issue. Research on parenting, in particular feeding, has focused on mothers (Davison et al., 2016; Morgan et al., 2017). To a large extent mothers still have the main responsibility for child feeding, which is an important expression of parenting (Pratt, Hoffmann, Taylor, & Musher-Eizenman, 2017), and this was shown in the ML study sample (Somaraki et al., 2020). However, increasing paternal participation in child raising has informed novel research focusing on fathers (Davison, Charles, Khandpur, & Nelson, 2017; Jansen, Harris, Daniels, Thorpe, & Rossi, 2018; Morgan et al., 2019). To this end, the ML study (which informs study II in the present paper) has purposefully involved both parents (caregivers) and assessed the parenting practices of both mothers and fathers (Ek et al., 2015). Nevertheless, a relatively recent study in Australia provides preliminary evidence that mothers and fathers have a similar understanding of an instrument assessing parenting in the feeding context (Jansen, Harris, Mallan, Daniels, & Thorpe, 2018). Moreover, fathers are consistent with mothers in their parenting styles (Kuppens & Ceulemans, 2019). This point has now been addressed in the text (pp. 30-31, lines 600-604).<br><br>The text reads:<br>*The greater participation of mothers in the validation study (almost 82%) compared to fathers may relate to mothers' primary feeding responsibilities [69]. However, recent studies have called for the inclusion of fathers in parenting research, and future research should evaluate similarities and differences between mothers' and fathers' parenting and feeding practices [56, 68, 75, 76].* (pp. 30-31, lines 600-604)
R2 Results study I	P. 17 Two parenting scales, limit setting and emotion regulation, confirmed factor loadings >0.4. However, these findings differ from the previously stated findings on P. 8, indicating that only monitoring and limit setting scales met the recommended levels. Please clarify the difference.
Author response	Thank you for giving us the opportunity to clarify. The recommended levels, which monitoring and limit setting reached, refer to the scale Content Validity Index (S-CVI), which was calculated based on the ratings by seven experts in child health care. The S-CVI was used to assess the content validity of the new parenting questionnaire at the earlier stages of the questionnaire development. In other words, it was used as an indicator of whether the developing questionnaire included relevant items, as deemed by experienced professionals in child health care. This point has now been clarified in pp. 9-10, lines 140-147.<br><br>The text reads:<br>*Seven experts in child health care, pediatrics, and pediatric psychology rated the 38 items based on how well they reflected the parenting skills of interest. The respective Content Validity Indexes on the sub scale level were computed (S-CVI) [36]. Subscales were deemed to measure what they intended to (by the child health care experts) if they included enough items relevant for assessing aspects of the corresponding parenting practice, meaning that on the subscale level, only items which the experts considered relevant were retained,*

	contributing to the calculation of the S-CVI. Only monitoring (0.92) and limit setting (0.96) reached the recommended level (S-CVI>0.90) [36]. (pp. 9-10, lines 140-147)<br><br>However, the factor loadings are linked to the validation of the finalized parenting questionnaire (which retained 9 items after consequent rounds of factor analysis), which included relevant items based on consecutive steps of instrument development, i.e. literature search, face/content validity (consulting health professionals) and cognitive interviews (consulting parents), as outlined in the Methods section. This point has been clarified in pp. 8-9, lines 117-121 and p. 15, lines 288-290.<br><br>The text reads:<br>*The development included the following stages: literature search and face validity (performed by the research group), content validity (consulting experienced professionals in child health care) and cognitive interviews (consulting parents). Once a pool of relevant items was constructed, the validation of the questionnaire consisted of the identification of patterns (subscales or factors) between the questionnaire items.* (pp. 8-9, lines 117-121)<br><br>The text reads:<br>*PCA with varimax normalized rotation (factors are not allowed to correlate) as well as with direct oblimin rotation (factors are allowed to correlate) was run on all 12 items, and the threshold for factor loading was set to 0.4.* (p. 15, lines 288-290)
R2 Results study I	P. 19 Were relevant covariate controlled for in the correlation analyses with parenting scales? This is important given the evidence at both the parent and child levels.
Author response	In identifying correlations between the new parenting questionnaire and other parenting scales, which are widely used in the field of childhood obesity, we did not attempt to identify pure associations and remove confounders. Rather, as child feeding is an expression of parenting, we expected both to correlate with parent and child background characteristics. We performed the correlations between the new parenting questionnaire and the other instruments in order to confirm that the new questionnaire captures relevant aspects of parenting, as expressed in the child feeding and obesity-related context, but still assesses some unique aspects of general parenting, i.e. limit setting and emotional regulation, which relate to child feeding, yet are not equivalent to it. In other words, we were interested in identifying correlations that were not too strong (meaning that the new instrument would assess the exact same thing as existing feeding instruments and therefore it would not offer additional information on parenting), and not too weak (meaning that the new instrument would not be relevant according to a "micro theory" that connects overall parenting and child feeding) (Kremers et al., 2013; Sleddens et al., 2014; van der Horst & Sleddens, 2017).
R2 Results study II	P. 19 Please define how 'foreign background' was assessed? Were they expected to differ in parenting or child outcomes? This should be introduced earlier with a rationale.

Author response	This point has now been addressed in the text (pp. 5-6 lines 41-52, p. 14 lines 261-265 and p. 31 lines 621-624).<br><br>The text reads:<br>*The ML study compares a parenting program with and without booster sessions (follow-up phone calls provided after the program, averaging 4 phone calls per family) and standard treatment; the primary study outcome is change in child weight status (body mass index standard deviation score, BMI SDS). The primary findings at 1-year post-baseline showed a greater decrease in child weight status among families randomized to the parenting program with booster sessions, compared to standard care and the parenting program without booster sessions [24]. In addition, the ML study includes a diverse sample with a high proportion of parents reporting foreign background (parent and/or their parents born outside Sweden) and lower educational attainment, unlike the majority of studies in the field which have included homogeneous samples [25]. Parental foreign background moderated treatment effects; specifically, boosters were necessary for sustained treatment effects among children whose parent(s) had foreign background [24].* (pp. 5-6 lines 41-52)<br><br>The text reads:<br>*In addition, parents (mothers and fathers separately) reported their age, weight, and height (to calculate BMI), level of education (with/without university degree) country of birth and their parents' country of birth, which further informed the categorization of parents (mothers/fathers) according to their foreign background (parent and/or their parents born outside Sweden).* (p. 14 lines 261-265)<br><br>The text reads:<br>*Future research should highlight and thoroughly examine the moderating role of migrant background in the effectiveness of childhood obesity treatment, as shown in relation to child weight status in the ML study [24], but also in relation to secondary outcomes, such as parenting practices.* (p. 31 lines 621-624)
R2 Results study II	P. 20 Were the linear mixed models adjusted for relevant covariates?
Author response	The linear mixed models were not adjusted for relevant covariates due to the study's design as a randomised controlled trial, where the three treatment groups differ in terms of the treatment approach offered to the families, who are otherwise randomised. Therefore, within each group the composition of families is similar and comparable so that the effects of treatment (the point that differentiates across groups) are examined, which, in the case of this paper, refer to secondary outcomes, i.e. parenting practices. This point has been clarified (p. 16, lines 313-314).<br><br>The text reads:<br>*The models were not adjusted for covariates due to the randomized design of the ML study.* (p. 16, lines 313-314)
R2 Results study II	P. 21 What were the estimates for the direct effects of parenting behaviors or treatment effects on BMI SDS in the mediation models? Did mediation models adjust for relevant covariates?

Author response	Please see our response to the previous comments in relation to the adjustment for relevant covariates in a randomised controlled trial. The mediation models were fitted in order to estimate the indirect effect of treatment on changes in child BMI SDS through changes in parenting practices. In this context, the direct effects are only interesting to the extent that they facilitate the calculation and understanding of indirect effects (Hayes & Rockwood, 2017). Otherwise, the direct effects of treatment on the primary outcome, i.e. changes in child weight status, have been examined in a recent publication by our group (Ek et al. (2019)), which is widely cited throughout the manuscript. Moreover, the direct effect of parenting behaviours on child BMI SDS was examined in the context of mediation models whereby the indirect effects of treatment effects on changes in child BMI SDS through changes in parenting practices were consistent with the aims of the manuscript and, therefore, the scope of the study. This point has been clarified in the Methods section (p. 17, lines 318-321).<br><br>The text reads:<br>*The PROCESS macro for SPSS, version 3.4.1 [54], was utilized to fit the mediation models and estimate the indirect effects of treatment on changes in child weight status through parenting practices (Fig 2), using 10 000 bootstrap samples to define Confidence Intervals (CIs) at the 95% level.* (p. 17, lines 318-321)
R2 Discussion	Overall, the discussion taps into several different areas. However, the explanations about the RCT effects should be modified. For example, this study found that mothers increased limit-setting overall, but not as a result of the intervention. This suggests that there was either a naturally-occurring increase in behaviors overtime or a third modifying variable. As for fathers, emotion regulation significantly changed for fathers in the two parenting intervention groups, assuming that the standard treatment was used as the comparison group (not a parenting intervention aimed at similar components of parenting as the other groups). However, this change indicated that fathers in the treatment group had a decline in emotion regulation (parenting tx = poorer emotion regulation). That said, no causal pathways were supported.
Author response	We agree with the points made by the reviewer. We modified the text to highlight the overall increase in maternal limit-setting (p. 28, lines 541-543).<br><br>The text reads:<br>*Whereas fathers' emotional regulation differed between standard treatment and the parenting program, mothers overall improved their ability to set limits, regardless of treatment condition.* (p.28, lines 541-543)<br><br>Moreover, we further modified the text to explain the possibility of a naturally-occurring increase in maternal limit setting (p.28, lines 545-547).<br><br>The text reads:

	These findings may imply that positive aspects of parental control increase during obesity treatment as part of applying lifestyle changes. (p.28, lines 545-547)<br><br>We believe that the explanation we have already provided in the Discussion (p. 28, lines 543-549, "*Similar findings were reported by Magarey et al. [67], who showed that integrating parenting components into childhood obesity treatment was as effective in increasing positive aspects of parenting as treatment focusing on lifestyle changes alone. These findings may imply that positive aspects of parental control increase during obesity treatment as part of applying lifestyle changes. However, this earlier study primarily involved mothers of older children and used a different tool to assess parenting practices. On average, mothers in our study attended more sessions of the parenting program, compared to fathers.*") aligns with the second point made in this comment, and the identification of other modifying variables, which may relate to the engagement to treatment and not the parenting program ("intervention") per se.<br><br>Regarding fathers' emotional regulation, we agree that our findings were contrary to the hypotheses, which we mention in Discussion (p. 27, lines 516-517, *"In study II, evaluation of changes in parenting practices in the ML study did not confirm our hypotheses."*), and it is true that findings regarding fathers were in an unexpected direction (p. 27, lines 519-520, *"Fathers' treatment group allocation was associated with changes in their capacity to regulate their own emotions, but in an unexpected direction"*). However, we found certain differences between the treatment groups regarding fathers' emotional regulation. Considering the randomized controlled design of the ML study, which provided the data on these secondary outcomes (mothers' and fathers' parenting practices), we believe that we have identified a causal link between treatment and fathers' emotional regulation, based on the current findings. We do not deny that there might be other factors contributing to these findings and in our Discussion we further explore and elaborate on possible explanations, for example the co-occurrence of limit setting and emotional regulation should be considered, since the limit setting practices of fathers did not change (p. 28, lines 531-534, *"Our findings suggest that weaker effects on paternal emotional regulation in the parenting program (decreased use), compared to standard treatment (increased use), may have facilitated child weight loss, since limit-setting practices remained consistent over time"*). In addition, in the discussion we compare and contrast the separate (and different) findings for mothers and fathers, since studies conducted within the last decade suggest that a more systemic approach to parenting, involving all caregivers at the same time is warranted (Gevers, van Assema, Sleddens, de Vries, & Kremers, 2015; Kuppens & Ceulemans, 2019).<br><br>The text in Discussion explaining the findings regarding maternal limit setting according to the points made in this comment:<br>*On average, mothers in our study attended more sessions of the parenting program, compared to fathers. Based on our own clinical experience and reports from primary health care nurses, we can assume that the situation is similar in standard treatment, though we did not collect these data. Taken together, these findings suggest that parallel improvements in maternal parenting regardless of treatment condition and intensity (parenting program, with/without boosters and standard treatment), may be explained by higher maternal engagement in treatment overall.* (pp. 28-29, lines 548-554)

R2 Discussion	P. 27, ln. 539 The authors suggest that this study addresses limitations in obesity research by using a more diverse SES sample. This is an overly ambitious statement given the limited details about the socio-demographic characteristics of the sample provided in the introduction and results. Additionally, no statistical testing (e.g., moderation) was performed for any conclusions about the differences of these various groups.
Author response	We agree with the reviewer that a mechanistical understanding of obesity treatment effectiveness requires moderation testing (which constitutes an interaction testing on statistical terms). This has been performed in the paper by Ek et al. (2019) in relation to the primary outcome, i.e. changes in child BMI SDS, which showed that fathers' foreign background is a relevant moderator. Therefore, we have added this point in the Introduction (pp. 5-6, lines 47-52) and Methods (p.14, lines 261-265). In addition, we highlight the strength of the heterogeneous ML sample in terms of foreign background and lower education attainment in the Discussion (p. 31, lines 614-617). However, the focus in study II has been on the mediation aspect of the mechanistic understanding of the ML study, as outlined in the aims.<br><br>The text in Introduction reads:<br>*In addition, the ML study includes a diverse sample with a high proportion of parents reporting foreign background (parent and/or their parents born outside Sweden) and lower educational attainment, unlike the majority of studies in the field which have included homogeneous samples [25]. Parental foreign background moderated treatment effects; specifically, boosters were necessary for sustained treatment effects among children whose parent(s) had foreign background [24].* (pp. 5-6, lines 47-52)<br><br>The text in Methods reads:<br>*In addition, parents (mothers and fathers separately) reported their age, weight, and height (to calculate BMI), level of education (with/without university degree) country of birth and their parents' country of birth, which further informed the categorization of parents (mothers/fathers) according to their foreign background (parent and/or their parents born outside Sweden).* (p.14, lines 261-265)<br><br>The text in Discussion reads:<br>*In addition, more than half of the parents participating in the ML study were first- or second-generation migrants. The sample had an overall lower educational attainment than the more homogeneous and well-educated samples dominating research in this field.* (p. 31, lines 614-617)
R2 Discussion	P. 27 Why was the power analysis only provided for treatment effects on child BMI SDS? Particularly since none of this study's aims/hypotheses examined the direct effect of treatment on child weight status.
Author response	The power analysis was based on the primary outcome, namely changes in child BMI SDS. This has now been explained in Methods (pp. 13-14, lines 240-246). In addition, in the Introduction (pp. 5-6 lines 38-50) we have elaborated on the ML study, its design and outcomes, as well as the findings on the primary outcomes. Based on these, the issue of power is included under limitations, since the study may have been underpowered to detect any significant changes regarding secondary outcomes, such as parenting practices (p. 31, lines 625-

	626, *"The study's primary limitation is that power calculations were based on the primary outcome of the ML study (changes in child weight status over a 12-month follow-up)"*).<br><br>The text reads:<br>*Sample size: The sample size calculation for the ML study was based on data from a treatment study in Germany [51]. On the basis of power calculations, 75 children were needed in each treatment (parent-only and ST adjusted for dropout) to detect a difference of 0.3 BMI z score (0.5 SD) with 85% power at 12 months' follow-up. The calculations included an adjustment for a dropout rate of 21%, based on data from a similar study of obesity treatment focusing on parenting [52]. The sample size calculation has been described previously [24].* (pp. 13-14, lines 240-246)<br><br>The text reads:<br>*The present paper attempts to address this gap in the literature by evaluating parenting practices and their effects on child weight status during a 12-month follow-up of an RCT for obesity treatment among preschoolers, the More and Less study (ML study) in Sweden [23]. The ML study compares a parenting program with and without booster sessions (follow-up phone calls provided after the program, averaging 4 phone calls per family) and standard treatment; the primary study outcome is change in child weight status (body mass index standard deviation score, BMI SDS). The primary findings at 1-year post-baseline showed a greater decrease in child weight status among families randomized to the parenting program with booster sessions, compared to standard care and the parenting program without booster sessions [24]. In addition, the ML study includes a diverse sample with a high proportion of parents reporting foreign background (parent and/or their parents born outside Sweden) and lower educational attainment, unlike the majority of studies in the field which have included homogeneous samples [25].* (pp. 5-6 lines 38-50)
R2 Minor points	In the methods and results, how the content of study 1 and 2 are presented is somewhat confusing to follow. Instead of switching back and forth between studies within each section, it may be easier to first present study 1 (methods, data analyses, and results) and then study 2 (methods, data analyses, and results).
Author response	We thank the reviewer for this valuable comment. We agree with the reviewer that the content of studies 1 and 2 is not easy to follow. However, we think it would be confusing to not follow the format "methods, data analyses, and results" for the paper. Therefore, we modified and now we refer to the manuscript as "paper" (p. 6, line 69), and throughout the text, "sub study I" and "sub study II" have been replaced by "study I" and "study II", respectively. We believe that this amendment clarifies the sections describing each study under the general subheading "methods, data analyses, and results".
R2 Minor points	In figure 3, the PGB and ST lines should reflect more distinguished differences (e.g., change one to a shade of gray or thicker lines).
Author response	Thank you for this suggestion. The three treatment groups are indeed illustrated using three distinct lines, which are also defined in the accompanying guide. Considering the present comment –and the absence of similar comments by the other reviewers or the editor–

	and given that different lines and shapes were used for the construction of the graph, within the capacity of the software used, we have not modified the figure.

Reviewer 3 (R3)

R3 Comments to the Authors	This manuscript concerns the validation of a new questionnaire about the evaluation of mothers' and fathers' parenting practices in relation to child weight status during a 12-month childhood obesity treatment trial. The manuscript is well-written, however, there are some points that need clarifications or changes. Please see my comments below.
Author response	We thank the reviewer for their suggestions.
R3 Abstract	In the abstract, the authors stated that the validation of the new questionnaire (12 items; responses on a 5-point Likert scale) revealed two dimensions of parenting (Cronbach's alpha ≥0.7). However, in the section of the results, they stated that the PCA and Cronbach's alpha calculations were done after three items were dropped from the 12-item questionnaire. I wonder if the final questionnaire contains 12 or 9 questions. This is a point that should be clarified and corrected.
Author response	Thank you for giving us the opportunity to clarify. We have now revised the abstract so that it shows the final composition of the questionnaire, which includes 9 items (p.2).<br><br>The revised text in the abstract reads:<br>*[…] The validation of the new questionnaire (9 items; responses on a 5-point Likert scale) revealed two dimensions of parenting (Cronbach's alpha ≥0.7) […]*
R3 Introduction	My major objection is the necessity for the construction of a new tool. At the end of the Introduction the authors just mentioned that "To evaluate parenting practices, a valid tool was needed [27]". Then they continued with "Therefore, a new parenting questionnaire, which could match the components of the ML parenting program, was developed". From this last sentence, it is deduced that this questionnaire was constructed just to meet the needs of the ML program. This point needs to be clarified as if this is the case, then the generalizability of the new tool is questioned.
Author response	We agree with the reviewer, and therefore we modified the text in Introduction (p. 6, lines 63-67) and Discussion (pp. 24-25, lines 451-454) so that it becomes clear that the development of the new parenting questionnaire is inextricably linked to the evaluation of parenting practices within the ML study in Sweden. In addition, we address the issue of generalizability of the new parenting questionnaire in the section on limitations (p. 31, lines 612-614).<br><br>The text reads:<br>*To evaluate the central parenting components of the ML program (encouragement, monitoring, limit-setting strategies, positive involvement, problem solving, and emotional regulation) a valid user-friendly tool was required. Given the lack of appropriate*

	evaluation instruments, we developed a questionnaire to assess changes in parental behaviors after participating in the ML program. (p. 6, lines 63-67)<br><br>The text reads:<br>*A new questionnaire on parenting practices, "Emotions and Communication in Parenting" (ECoP), was developed and validated to evaluate the evidence-based parenting practices included in the ML parenting program.* (pp. 24-25, lines 451-454)<br><br>The text reads:<br>*However, further research is needed to establish the relevance of the ECoP in relation to child developmental outcomes other than childhood obesity, which has been the focus in the present paper.* (p.31, lines 612-614)
R3 Materials and Methods	The literature search procedure needs more description. The authors should report all databases they searched. Furthermore, it is not clear whether only 14 questionnaires were identified or were more tools and the authors selected these 14?
Author response	The text has been modified based on the suggestions in the comment (p. 9, lines 123-124, p.9, lines 127-129).<br><br>The text reads:<br>*To identify papers describing parenting questionnaires, Google, Google Scholar, PubMed, Sirus and Web of Science were searched.* (p. 9, lines 123-124)<br><br>The text reads:<br>*This resulted in the collection of 14 questionnaires and 396 items (in English and Swedish), which were deemed relevant to evaluating the practices addressed in the ML program.* (p.9, lines 127-129)
R3 Materials and Methods	By what sampling method were the fifteen schools and thirty preschools selected?<br>In the school sample, almost half of the parents (431 out of 931) returned completed questionnaires. The authors should comment on whether this has any effect on the results. Do the characteristics of the parents who did not respond differ from those of the parents who participated?
Author response	The schools and preschools were sampled to represent areas of varied obesity prevalence, as explained in the paper (p.11, lines 177-179). Regarding the response rate in the school/preschool sample (431 out of 931, i.e. 46.3%), it is similar to other studies examining parenting in the field of childhood obesity. Although a certain bias had been introduced (i.e. well-educated sample) the addition of the clinical sample increased the diversity of the total sample. This information and a comment on the external validity of the study has been clarified under limitations in the Discussion (p. 31, lines 604-608).<br><br>The text reads:

	Although the sample in the validation study was well-educated and with a lower proportion of parents with a foreign background compared to the general population, the response rate (46%) was similar to earlier studies focusing on parenting instruments [43, 77]. The addition of the clinical sample yielded a more heterogeneous population, increasing the external validity of the study. (p. 31, lines 604-608)
R3 Results	The "Child obesity" variable displayed in Table 1 has not been defined. How were the children categorized as obese and not obese? In general, all variables used in this study should be mentioned and explained.<br>In line 336 the authors stated that on average, mean scores on LS were higher than scores on ER (4.03 vs. 3.51). They should also provide the corresponding p-value.
Author response	We agree with the reviewer that the variables characterising the sample (background characteristics of the parents and the child) need to be clearly defined. We have now modified the text accordingly (pp. 11-12, lines 188-199, p. 14, lines 259-266). We have also added the corresponding p-value (p. 21, line 388).<br><br>The text reads:<br>*Covariates/background questionnaires: Child BMI SDS was based on parent-reported data on child height and weight for the school sample and on measured data for the clinical sample. Calculations of the BMI SDS and the classification of children according to their obesity status (children with obesity and without obesity) –in line with the focus of this paper on childhood obesity per se– were based on the criteria by Cole and Lobstein (41). Moreover, 18 children were classified in the underweight category, according to age- and gender-specific criteria for thinness among children (child weight status equivalent to BMI<17) [42], and they were included in the non-obesity category. Parents' heights and weights were self-reported and used to calculate parental Body Mass Index (BMI= kg/m2). Parents also reported on their education level (further categorized into university degree or no university degree) and their country of birth. Moreover, data on child age and gender were parent-reported in the school sample and were made available upon referral of children in the clinical sample.* (pp.11-12, lines 188-199)<br><br>The text reads:<br>*Covariates/background characteristics: Background questionnaires were filled out by parents, who reported on family structure (birth order of the child and if the child lived with both parents or not). In addition, parents (mothers and fathers separately) reported their age, weight, and height (to calculate BMI), level of education (with/without university degree) country of birth and their parents' place of country of birth, which further informed the categorization of parents (mothers/fathers) according to their foreign background (parent and/or their parents born outside Sweden). Information on child's age and gender were provided upon referral from healthcare.* (p. 14, lines 259-266)
R3 Discussion	In lines 409 – 413, the authors stated that although they expected the new questionnaire to capture all the parenting practices addressed in the ML study, it captured only two of the dimensions of parenting (parents' capacity to set limits to the child and parents' capacity regulate their 413 own emotions in parenting situations). I would expect to see some possible explanations for this.

Author response	We agree with the reviewer that this is a relevant point, which warrants further discussion. We restructured the text following the statement addressed in this comment, so that a possible explanation is offered (pp. 25-26, lines 464-482).<br><br>The text reads:<br>*We expected the questionnaire to capture all the parenting practices addressed in the ML program (monitoring, positive involvement, limit setting, problem solving, emotional regulation, and encouragement). However, after consulting an expert group and parents, we found the final questionnaire identified two key dimensions of parenting, both of which allowed us to assess variations in parental practices: 1) parents' capacity to set limits to the child and 2) parents' capacity to regulate their own emotions in parenting situations. The findings may suggest that these two dimensions of parenting better differentiate between parents in the Swedish context (according to health professionals and parents), compared to the dimensions not represented in the final 9-item questionnaire. Interestingly, the two dimensions map onto the overarching dimensions that define parenting styles, i.e., responsiveness and demandingness [55-58]. Setting limits to one's child corresponds to providing clear and consistent boundaries and structure, while being able to regulate one's own emotions when interacting with the child allows parents to be responsive to the child's needs. Thus, it may be possible that the remaining dimensions of parenting which are practiced during the ML group sessions – i.e. positive involvement, monitoring, encouragement, and problem solving – act together to influence overarching dimensions of parenting (demandingness and responsiveness). This suggests that, at least in Sweden, all dimensions may be best assessed through parental limit setting and emotional regulation strategies.*

Buscot, M. J., Thomson, R. J., Juonala, M., Sabin, M. A., Burgner, D. P., Lehtimaki, T., . . . Magnussen, C. G. (2018). BMI Trajectories Associated With Resolution of Elevated Youth BMI and Incident Adult Obesity. *Pediatrics, 141*(1). doi:10.1542/peds.2017-2003

Croker, H., Cooke, L., & Wardle, J. (2011). Appetitive behaviours of children attending obesity treatment. *Appetite, 57*(2), 525-529. doi:10.1016/j.appet.2011.05.320

Davison, K. K., Charles, J. N., Khandpur, N., & Nelson, T. J. (2017). Fathers' Perceived Reasons for Their Underrepresentation in Child Health Research and Strategies to Increase Their Involvement. *Matern Child Health J, 21*(2), 267-274. doi:10.1007/s10995-016-2157-z

Davison, K. K., Gicevic, S., Aftosmes-Tobio, A., Ganter, C., Simon, C. L., Newlan, S., & Manganello, J. A. (2016). Fathers' Representation in Observational Studies on Parenting and Childhood Obesity: A Systematic Review and Content Analysis. *Am J Public Health, 106*(11), e14-e21. doi:10.2105/ajph.2016.303391

Dishion, T., Forgatch, M., Chamberlain, P., & Pelham, W. E., 3rd. (2016). The Oregon Model of Behavior Family Therapy: From Intervention Design to Promoting Large-Scale System Change. *Behav Ther, 47*(6), 812-837. doi:10.1016/j.beth.2016.02.002

Ek, A., Chamberlain, K. L., Ejderhamn, J., Fisher, P. A., Marcus, C., Chamberlain, P., & Nowicka, P. (2015). The More and Less Study: a randomized controlled trial testing different approaches to treat obesity in preschoolers. *BMC Public Health, 15*, 735. doi:10.1186/s12889-015-1912-1

Ek, A., Lewis Chamberlain, K., Sorjonen, K., Hammar, U., Etminan Malek, M., Sandvik, P., . . . Nowicka, P. (2019). A Parent Treatment Program for Preschoolers With Obesity: A Randomized Controlled Trial. *Pediatrics, 144*(2). doi:10.1542/peds.2018-3457

Gevers, D. W., van Assema, P., Sleddens, E. F., de Vries, N. K., & Kremers, S. P. (2015). Associations between general parenting, restrictive snacking rules, and adolescent's snack intake. The roles of fathers and mothers and interparental congruence. *Appetite, 87*, 184-191. doi:10.1016/j.appet.2014.12.220

Hayes, A. F., & Rockwood, N. J. (2017). Regression-based statistical mediation and moderation analysis in clinical research: Observations, recommendations, and implementation. *Behav Res Ther, 98*, 39-57. doi:10.1016/j.brat.2016.11.001

Jansen, E., Harris, H., Daniels, L., Thorpe, K., & Rossi, T. (2018). Acceptability and accessibility of child nutrition interventions: fathers' perspectives from survey and interview studies. *Int J Behav Nutr Phys Act, 15*(1), 67. doi:10.1186/s12966-018-0702-4

Jansen, E., Harris, H. A., Mallan, K. M., Daniels, L., & Thorpe, K. (2018). Measurement invariance of the Feeding Practices and Structure Questionnaire-28 among a community of socioeconomically disadvantaged mothers and fathers. *Appetite, 120*, 115-122. doi:10.1016/j.appet.2017.08.030

Kremers, S., Sleddens, E., Gerards, S., Gubbels, J., Rodenburg, G., Gevers, D., & van Assema, P. (2013). General and food-specific parenting: measures and interplay. *Child Obes, 9 Suppl*(Suppl 1), S22-31. doi:10.1089/chi.2013.0026

Kuppens, S., & Ceulemans, E. (2019). Parenting Styles: A Closer Look at a Well-Known Concept. *Journal of child and family studies, 28*(1), 168-181. doi:10.1007/s10826-018-1242-x

Morgan, P. J., Collins, C. E., Lubans, D. R., Callister, R., Lloyd, A. B., Plotnikoff, R. C., . . . Young, M. D. (2019). Twelve-month outcomes of a father-child lifestyle intervention delivered by trained local facilitators in underserved communities: The Healthy Dads Healthy Kids dissemination trial. *Transl Behav Med, 9*(3), 560-569. doi:10.1093/tbm/ibz031

Morgan, P. J., Young, M. D., Lloyd, A. B., Wang, M. L., Eather, N., Miller, A., . . . Pagoto, S. L. (2017). Involvement of Fathers in Pediatric Obesity Treatment and Prevention Trials: A Systematic Review. *Pediatrics, 139*(2). doi:10.1542/peds.2016-2635

Pratt, M., Hoffmann, D., Taylor, M., & Musher-Eizenman, D. (2017). Structure, coercive control, and autonomy promotion: A comparison of fathers' and mothers' food parenting strategies. *J Health Psychol*, 1359105317707257. doi:10.1177/1359105317707257

Sleddens, E. F. C., O'Connor, T. M., Watson, K. B., Hughes, S. O., Power, T. G., Thijs, C., . . . Kremers, S. P. J. (2014). Development of the Comprehensive General Parenting Questionnaire for caregivers of 5-13 year olds. *International Journal of Behavioral Nutrition and Physical Activity, 11*(1), 15. doi:10.1186/1479-5868-11-15

Somaraki, M., Eli, K., Sorjonen, K., Ek, A., Sandvik, P., & Nowicka, P. (2020). Changes in parental feeding practices and preschoolers' food intake following a randomized controlled childhood obesity trial. *Appetite, 154*, 104746. doi:https://doi.org/10.1016/j.appet.2020.104746

van der Horst, K., & Sleddens, E. F. C. (2017). Parenting styles, feeding styles and food-related parenting practices in relation to toddlers' eating styles: A cluster-analytic approach. *PLoS One, 12*(5), e0178149. doi:10.1371/journal.pone.0178149

Webber, L., Cooke, L., Hill, C., & Wardle, J. (2010). Associations between children's appetitive traits and maternal feeding practices. *J Am Diet Assoc, 110*(11), 1718-1722. doi:10.1016/j.jada.2010.08.007
