# Supplementary material for: Parenting and childhood obesity: Validation of a new questionnaire and evaluation of treatment effects during the preschool years
Source: PLoS One. 2021 Sep 23;16(9):e0257187. doi: 10.1371/journal.pone.0257187 (PMC8459975; doi:10.1371/journal.pone.0257187)
Supplement: Attachment — Submitted filename: renamed_09901.docx [file pone.0257187.s006.docx]

**COMMENTS – Manuscript PONE-D-20-28940**

Title: “Parenting and childhood obesity: Validation of a new questionnaire and

evaluation of treatment effects during the preschool years”

**Important note: This review pertains only to ‘statistical aspects’ of the study and so** **‘****clinical aspects’ [like medical importance,** **relevance of the study,** **‘clinical significance and** **implication(s)’ of the whole study, etc.] are to be evaluated [should be assessed] separately/independently. Further please note that any ‘statistical review’ is generally done under the assumption that (such) study specific methodological [as well as execution] issues are perfectly taken care of by the investigator(s). This review is not an exception to that and so does not cover clinical aspects {however, seldom comments are made only if those issues are intimately / scientifically related & intermingle with ‘statistical aspects’ of the study}. Agreed that ‘statistical methods’ are used as just tools here, however, they are vital part of methodology [and so should be given due importance].**

**COMMENTS:** Your ABSTRACT is well drafted but assay type. Please note that it is preferable [refer to item 1b of CONSORT checklist 2010: Structured summary of trial design, methods, results, and conclusions] to divide the ABSTRACT with small sections like ‘Objective(s)’, ‘Methods’, ‘Results’, ‘Conclusions’, etc. which is an accepted practice of most of the good/standard journals [including PLOS-ONE]. It will definitely be more informative then, I guess, whatever the article type may be.

Unfortunately, there are several questions [naturally arising in mind] about this article. Few important/vital ones [not possible to cover all] are described/given below. First let us refer to lines 198-202, section on ‘Sample size’ [as vital for present statistical review] which quotes reference by Kleber et al., 2009 {‘Power calculations were based on the primary outcome, child BMI SDS (Body Mass Index Standard Deviation Score) (Kleber et al., 2009)}. Are you referring to reference number 19? But it is said in line 30 that their study (reference 19) is ‘without a control condition’ then how can ‘power calculations are based’ on this study or do you want to say that ‘the primary outcome, child BMI SDS (Body Mass Index Standard Deviation Score)’ this term is used for first time by them or the term is taken from this reference? Clarify the purpose of quoting the article. Note that “Kleber” is second author of paper quoted as 19 and instead of [19], quoting (Kleber et al., 2009) is not correct at all. Please follow the standard practice of quoting reference(s).

Further, it is said that ‘…. adjusting for a dropout rate of 21%’. Are following references (namely Ek, Chamberlain, et al., 2015; West et al., 2010) are for the (odd) figure of 21%? Account given in section on ‘Sample size’ (lines 198-202) ‘Seventy-five children needed to be included in each of the treatment approaches (ML program and standard treatment) in order to identify a difference in BMI SDS between the groups at 12-months post-baseline’ is/are inadequate [not sufficient at all] and therefore, of no use { to identify what amount of difference in BMI SDS?}.

Though measures/tools used are appropriate, most of them [example: Child Feeding Questionnaire (CFQ), the Lifestyle Behaviour Checklist (LBC), etc.] yield data that are in [at the most] ‘ordinal’ level of measurement [and not in ratio level of measurement for sure {as the score two times higher does not indicate presence of that parameter/phenomenon as double (for example, a Visual Analogue Scales VAS score or say ‘depression’ score)}]. Then application of suitable non-parametric test(s) is/are indicated/advisable [even if distribution may be ‘Gaussian’ (i.e. normal)].

Now refer to lines 228-234

{**Statistical Analyses - Sub study I:** Differences between subsamples were examined using independent samples t-test for continuous variables and chi-squared test for categorical variables. Background characteristics were compared across the school and clinical samples using one-way Analysis of Variance (ANOVA) (for continuous variables) and chi-square test (for categorical variables).}

Use Mann-Whitney U test in place of independent samples t-test. Note that we use Analysis of Variance (ANOVA) [for continuous variables] to compare three or more groups {not to compare two groups]. You seem to compare two groups [the school and clinical samples] only. Following note is from one standard text-book on Biostatistics:

When only two groups are to be compared {ex. Women and Men}, we use ‘t’ test for two independent groups [non-parametric equivalent to unpaired ‘t’ test is Mann-Whitney ‘U’ test] and not ANOVA ‘F’ Although ‘F’ and ‘t’ are mathematically related/equivalent [square of ‘t’ is exactly equal to ‘F’ if (mistakenly) calculated for two groups], logic/philosophy (and so underlying assumptions) behind their development and algorithms used for estimation of test statistic are different. Mind you that they are applicable in different situations.

Choice of test will not depend on ‘Type of variable’ or ‘Level of measurement’ of parameter/background characteristic [line 231 or 256] under consideration. {Non-parametric equivalent of one-way ANOVA ‘F’ is ‘Friedman’s test’}. If treatment group are now [i.e. your are considering] three conditions [as PGB: Parent group with booster sessions; PGNB: Parent group without booster sessions; ST: Standard Treatment], clarify. Kindly remember that this is a scientific/academic document and so all details should be clearly communicated.

In ‘Abstract’ you say “A merged school/clinical sample (n=558, 82% mothers) was used for the factorial and construct validation of a new parenting questionnaire. Changes in parenting were evaluated using data from the More and Less Study, a randomized controlled trial (RCT) with 174 children (mean age=5 years, mean BMI-Z =3.0) comparing a parent support program (with and without booster sessions) and standard treatment.”. Further, you say “We administered the new questionnaire to the RCT participants.”. Is not that confusing? Where and when this RTC (line 39-40: an RCT for obesity treatment among preschoolers, the More and Less study (ML study) [23].) was conducted? As said in lines 54-6 [The evaluation includes two stages: performing a validation study on a new questionnaire on parenting practices (Sub study I) and assessing parenting practices using data from the ML study RCT (Sub study II).] it seems that you have used entirely different samples for these two stages {Sub study I & Sub study II}. Is that true? It is not clear ‘whether n=558 or n=174’ for this study [how can different stages of the same can use different samples?]. Again, remember that this is a scientific/academic document and so all details should be clearly communicated. Do not confuse readers. I found many things confusing in this article. Please do not take readers for granted {that they will understand what is there in your mind}. It may be 100% correct, but need to (should) be communicated clearly.

Refer to Table 3 [Correlations between Limit Setting/Emotional Regulation and the CFQ and LBC. Mean group differences in Limit Setting/Emotional Regulation between children with obesity and children without obesity (normal weight/overweight)]. In this context note that [mainly because you have used ‘n.s.’ in this table],

Statistical test usually used to assess significance of Pearson’s ‘Correlation coefficient (r)’ is ‘t’ [where t = { r √ [(n-2) / (1-r^2^)] }for df=n-2, n is sample size] and here Ho is that the population/standard value of ‘r’ is zero. You need r=0.878 to be significant at 5% when n=5 but you need r=0.273 if n=50 & you need only r=0.088 if n=500. ‘P-value’ heavily depends on sample size. Therefore, it is customary to use the (available in most text books on ‘Biostatistics’ or on ‘www/net’) following guidelines for interpreting positive or negative correlations (and do not rely only on corresponding ‘P’-value but also consider an absolute value of ‘Correlation coefficient’). [This argument is equally applicable to non-parametric Spearman’s ‘Correlation coefficient (ρ)’ as well.]

There are two more questions regarding Table three.

1. You have tested mean group differences in ‘Limit Setting & Emotional Regulation’ between children with obesity and children without obesity separately by ‘Individual samples t-test’ which is perfectly alright but why ‘Paired samples t-test’ is applied for TOTAL? Is that correct (are you dealing with pairs now)? How? Please explain {this question is due to footnote}.

2. Why references are quoted in footnote [^a^ Validation study of the CFQ in Sweden [35], ^b^ Validation study of the LBC in Sweden [33]]. This implies that ‘r’ values are reported from these references. Is that so? Please explain.

I refrain from giving adverse comments on many other points in manuscript however, I definitely feel [am almost sure] that this study has potential. Presentation of (hard achieved) material is poorly done. Re-drafting avoiding confusion is recommended.
